# Finite element analysis and optimization of tractor gearbox body under various kinds of working conditions

**DOI:** 10.1038/s41598-022-22342-6

**Published:** 2022-10-17

**Authors:** Sihui Dong, Shiqun Li, Shenghui Fu, Kang Wang

**Affiliations:** 1 School of Traffic and Transportation Engineering, Dalian Jiaotong University, Dalian, 116028 China; 2 School of Mechanical and Electronic Engineering, Shandong Agricultural University, Tai’an, 271000 China; 3 College of Safety Science and Engineering, Xi’an University of Science and Technology, Xi’an, 710054 China

**Keywords:** Engineering, Mechanical engineering

## Abstract

As the main component of the tractor gearbox, the box has the functions of shifting operation and carrying the cab, it also carries part of the framework function. If the strength, stiffness, or vibration characteristics of the box do not meet the allowable requirements, large vibration and noise may occur, and even there is the possibility of fracture in some limiting conditions. To solve this problem, according to the structural parameters of a gearbox, the three-dimensional model of the box was established by using the three-dimensional modeling software Creo. According to the dangerous degree of the transmission load when the tractor is working normally, three vehicle working conditions are selected: a round of suspension conditions, farm tool lifting conditions, and emergency turning conditions. In addition, according to the transmission ratio of each gear meshing inside the gearbox, two gear conditions are selected: gear condition one and reverse gear condition one. The forces of the box under these extreme conditions are analyzed. The static analysis and modal analysis of the tractor gearbox are carried out by using the Static Structural module of ANSYS Workbanch. The deformation, equivalent stress distribution, and modal vibration frequency of the gearbox are tested. The topology optimization method is used to improve structural defects and reduce box quality. The results show that the weight of the optimized box reduces by 8.44%, the deformation decreased by 15.89%, and the equivalent stress decreased by 18.34%. The strength and stiffness of the box are improved, the quality is lightweight, the waste of resources is reduced, and the heat dissipation performance and fracture resistance of the box are enhanced.

## Introduction

In agricultural machinery, the tractor is the most important source of power output^1^. As the main part of the gearbox, the box needs to deal with a variety of extreme conditions, so the working environment is relatively poor^2^. When necessary, it may carry the cab and part of the frame, withstand a variety of external forces, and sometimes produce large vibration and noise, which affects the working quality of the driver. Therefore, the box must meet the requirements of strength, stiffness, and vibration characteristics. And based on ensuring the box foundation structure, we try to reduce the box quality and enhance the rationality of the box structure. How to comprehensively and effectively test the rationality of the gearbox structure and ensure the normal operation of the tractor gearbox has always been an important issue in the neighborhood of agricultural machinery. Therefore, it is necessary to consider effective detection methods and comprehensive and representative detection conditions. At present, the research on the structure design, detection and optimization of tractor gearboxes still has the problems of low efficiency and one-sided detection. Exploring representative limit conditions, and using finite element technology to analyze the strength, stiffness and vibrational characteristics of the box under these conditions, and then proposing optimization measures is a shortcut to solve the structural problems of the tractor gearbox. Weis et al. studied the modal characteristics of the gearbox body under external load, and used simulation software to determine whether the box meets the requirements^3^. Abbes and Fakhfakh proposed a finite element dynamic substructure method based on the generation of the overall noise of the gearbox to determine the natural frequency and the corresponding vibration mode of the box^4^. Aiming at the problem of vibration failure under the limiting conditions of gear, to avoid the damage and vibration of gear, Lim et al. proposed a gearbox structure, which makes the vibration frequency of the gearbox decrease^5^. The above results show that a good box structure can effectively avoid excessive vibration and noise. However, these methods focus on judging the vibration properties of the box. Whether the designed box meets the complex and changeable working conditions is not fully listed, and can not comprehensively solve the structural problem of the gearbox box. Sonsino has carried out the strength analysis of the gearbox body and proposed the gearbox body design method^6^. Ulu et al. proposed a series of structural optimization methods to solve the problem of the uncertainty of the stress position of the box structure, and it is quickly applied^7^. Tianfei Ma et al. analyzed the strength, stiffness, and fatigue life of the gearbox by multi-body dynamics method^8^. Kennedy et al. proposed gradient-based structural design and optimization methods^9^. Vasim Bashir Maner et al. designed and optimized the gearbox body of a vehicle^10^. Patil et al. optimized the differential gearbox housing^11^. Yang et al. carried out the simulation and dynamic test of the box, so that the mechanical properties of the box are more in line with the actual use^12^. The above results show that the good mechanical properties of the box can effectively avoid the fracture and deformation of the box, which is more important in ensuring the normal shift operation of the tractor gearbox and undertaking the vehicle. However, these methods directly or indirectly regard the detection of the loading condition of the box as the detection of the tensile stress or torsion of its material. To ensure that the strength and rigidity of the box structure meet the requirements, these methods are correct. However, considering the actual working conditions of the tractor, these methods still have the problem of incomplete consideration. Therefore, we conducted holistic detection and comprehensive research on the box to achieve the accuracy of the results. We analyzed a variety of working conditions of the tractor to be more in line with the actual working conditions of the tractors. One is the force given to the box by the internal gear train of the gearbox, that is, the two working conditions with the largest transmission ratio in the forward gear and the reverse gear are analyzed; the second is to analyze the force transmitted to the gearbox housing of the tractor due to the ground excitation during the normal operation of the tractor, as well as the acceleration, external load and frame torsion deformation of the tractor during operation. Here, we integrate these complex situations, collectively referred to as the vehicle condition of the gearbox. After actual investigation and experimental simulation, we studied the three working conditions of tractor front wheel suspension, emergency turning and suspension lifting, and collected a variety of complex stresses, such as the engine shell, front axle shell and rear axle shell connected to the gearbox housing. The torsional load of the housing and the external load are transmitted to the housing through the connector when the rear suspension farm tool is lifted, which greatly restores the real force of the housing. On this basis, the finite element analysis of the gearbox box is carried out to simulate the real force situation, not only considering a certain force. Therefore, the accuracy of the results is more in line with the actual requirements in theory.

Proposed by Kim et al., as one of the most effective tools for lightweight design, has been implemented in many industries and fields to enhance product development^13^. Leite et al. put forward the lightweight design method of vehicle automatic gearbox body. The research conclusions prove that this optimization method can be used in the early stage of box design^14^. Du Pengyu studied the structure and lightweight of power shift tractor transmission^2^. The above results show that the lightweight design of the box is necessary. Based on meeting the mechanical properties of the box, reducing the weight of the box can effectively save resources and enhance the heat dissipation performance. The above results show that box optimization is a necessary design. Therefore, we use ANSYS to detect the integrity of the gearbox. If the box can not meet the allowable requirements under the conditions of the whole vehicle and the gear train set by us, then the necessary structural optimization of the gearbox should be carried out to enhance the structural rationality of the tractor gearbox, and the topology optimization function should be used to realize the lightweight of the box, reduce the quality of the box, effectively save resources and enhance the heat dissipation performance, so that the whole research can be closed-loop.

At present, on the one hand, the static analysis and modal analysis of gearbox housing based on ANSYS often lack consideration of working conditions which can not fully reflect the actual stress situation, equivalent stress, and total deformation distribution of the gearbox body under ultimate state. On the other hand, the lightweight design of the box often ignores the strength and stiffness requirements of the box itself. According to the basic parameters of the gearbox body of a tractor, the gearbox body model is established by using three-dimensional modeling software. A variety of limit conditions are divided, and the stress of the gearbox housing under the limit conditions is calculated. On this basis, the finite element strength, stiffness, and modal analysis of the gearbox body are carried out, and then the gearbox body model is optimized to test whether the model meets the design requirements. It is conducive to improving the anti-deformation and anti-fracture ability of the box under the limiting conditions, and ensuring that no fracture occurs in the use process. It is conducive to reducing the weight of the box to achieve lightweight, reducing waste of resources, and enhancing the heat dissipation performance of the box. It can simulate and solve the possible failure of the box before practical application. According to the actual investigation into a variety of extreme conditions, calculate the force of the box under extreme conditions, which makes our research can better reflect the actual working conditions of the gearbox in the normal operation of the tractor, and then theoretically improve the accuracy of box simulation.

## Box modeling and simplification

### 3D modeling of box

**Figure 1:**
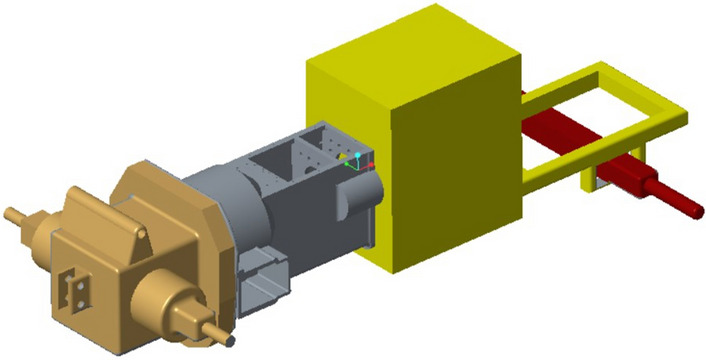
Transmission assembly diagram.

According to the structural parameters of a tractor gearbox, a three-dimensional model of the gearbox is established. The front end of the gearbox is connected to the engine, which is the power input end; the back end is connected with the back bridge, and the standard bolt M8X25-GB5783 is connected with the gasket 8-GB93. As is shown in Fig. 1.

**Figure 2:**
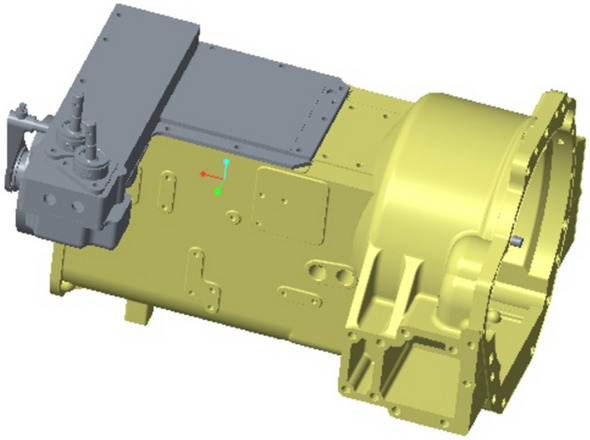
Transmission assembly diagram.

The maximum size of the gearbox housing is 785 mm × 543 mm × 596 mm. The gearbox body is equipped with various oil holes, inlet and outlet oil outlets, positioning pin holes, observation ports and other structures, and includes a large number of rounds and part of the surface modeling design. There are 52 bolt holes, 4 positioning pin holes, 4 stiffeners, 1 observation port, etc. As is shown in Fig. 2.

### Simplification of box model

The gearbox is large in volume and complex in structure. When ANSYS is used to analyze the structure of the gearbox, the original model is meshed, and each feature structure can generate a large number of nodes and units. Too small feature structure needs to be divided into small cells, so as to increase the amount of data processing, which may cause the analysis impossible ^15^. Moreover, the structures such as the oil outlet and the transition fillet of the box have no effect on the static analysis and modal analysis of the box. It is impossible that the mass matrix and stiffness matrix of the model are completely consistent with the actual situation when the finite element model of the gearbox is established. Therefore, it is necessary to simplify the structure according to the equivalent principle, and the simplified gearbox can be obtained under the condition of ensuring the high accuracy of the calculation results. The experiment proves that it has little effect on the final finite element analysis results. The following is the simplification of the gearbox:

1. The front box and the rear box are connected by bolts during installation. The bolt fastening force can be regarded as the internal force of the whole case. In the finite element analysis, the left and right transmission box is taken as a whole^16^.
2. The structures such as oil channel holes, integrated valve seats, oil outlets and rounds are removed, and the stiffeners on both sides and blind holes and thread holes with small diameters are removed.

**Figure 3:**
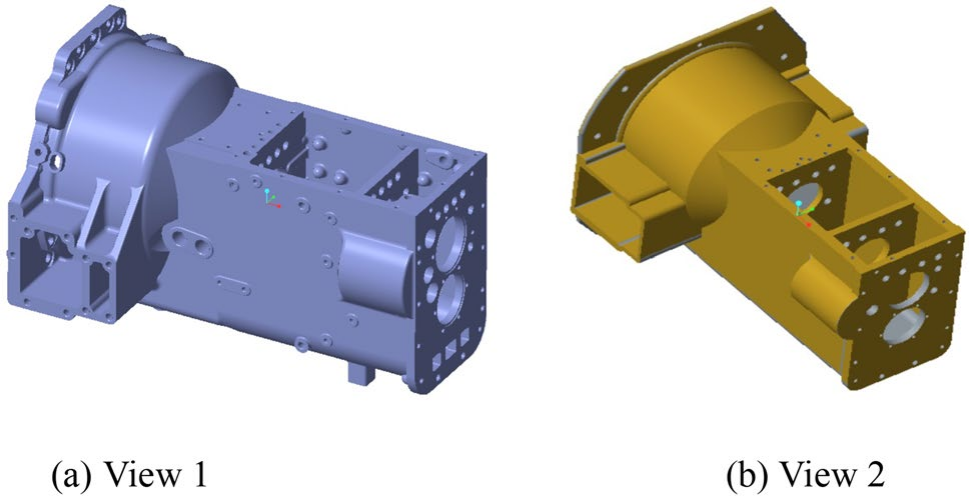
Comparison of the simplified box model.

These structures have little effect on the finite element analysis results, which can make the analysis more concise and the data more objective. The comparison of the simplified model of the box is shown in Fig. 3.

## Transmission condition analysis

As a part of the frame, the tractor gearbox bears different vector forces under different gearbox gears and vehicle conditions, resulting in different degrees of combined deformation of tension, bending and torsion^1^. To study the possible damage to the tractor gearbox under normal working conditions, we should analyze the extreme working conditions. The limit condition refers to the combination condition of the whole vehicle condition and the gear condition under the limit state of the tractor, which is a representative gearbox condition. The limit state of the tractor includes the dangerous state that the emergency turning of the vehicle under the large gear ratio or its round of suspension rotation may lead to the fracture of the gearbox, the bending deformation of the frame and even the rollover of the tractor. Therefore, the limit state of the tractor is dangerous and not unique, and the corresponding limit conditions are diverse, but the most representative vehicle conditions are emergency turning conditions, one-wheel suspension conditions, and farm tool lifting conditions: the shift condition is the forward one and the reverse one. The force analysis of the gearbox box under typical working conditions is carried out to determine the boundary conditions of finite element analysis.

### Vehicle condition analysis

**Figure 4:**
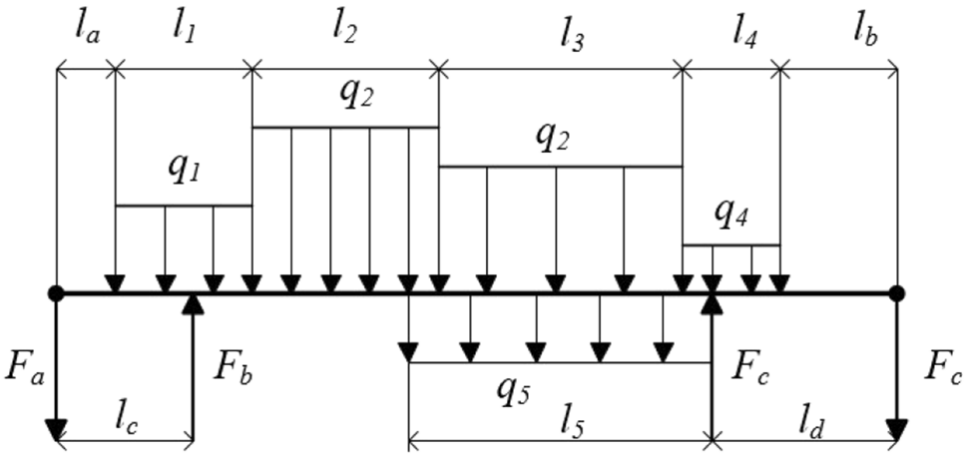
Simplified mechanical model of tractor.

Tractors in static and no load conditions, normal driving conditions on a flat road, uniform turning conditions, suspension lifting conditions, because the engine, gearbox and other transmission parts act as a frame, to bear the gravity of the parts, the box will naturally occur vertical bending deformation. The actual survey found that most of the tractor layout is symmetrical distribution, so we believe that there is no torsion deformation. The stress of the box is simulated in the form of a simply supported beam, and the mechanical model is shown in Fig. 4.

In Fig. 4, *F*_*a*_ represents the weight of the tractor front weight block and the front suspension weight, N; *l*_*a*_ represents the distance from the center of the front counterweight block to the counterweight frame, mm; *F*_*b*_, *F*_*c*_ represents the front bridge support, rear bridge support, N; *l*_*b*_ represents the distance from the rear suspension center to the rear axle, mm; *l*_*c*_ indicates the distance from the proposed front axle support point to the locomotive, mm; *l*_*d*_ represents the distance from the proposed rear axle support point to the locomotive, mm; *F*_*d*_ represents rear suspension lifting force, N; *q*_1_, *q*_2_, *q*_3_, *q*_4_, *q*_5_ represent the equivalent weight of front counterweight frame, engine, gearbox, rear axle, auxiliary structure (fuel tank, etc.), N/mm; *l*_1_, *l*_2_, *l*_3_, *l*_4_, *l*_5_ represent the length of front counterweight frame, engine, gearbox, rear axle and auxiliary structure, mm.

1. Tractor emergency turning condition analysis

Tractors may be working in emergency turn when operating in harsh conditions. When the tractor turns emergency, it will produce a large centripetal acceleration, here recorded as emergency acceleration. This is noted as emergency acceleration. The box and its front and rear connecting parts are affected by acceleration, resulting in bending deformation and lateral torsional deformation.

When the tractor encounters an emergency or the tire slips, the box has a large centripetal acceleration. The emergency turning radius is determined according to the minimum turning radius, and the emergency turning acceleration is calculated.

The minimum turning radius can be calculated by the formula (1) and formula (2).

1 $$R = \frac{L}{{2{\text{sin}}\psi }}$$

2 $$D = W + 2R \cdot (1 - {\text{cos}}\psi )$$

where *R* denotes the minimum turning radius, m; *L* represents the length, m; *W* represents vehicle width, m; *D* represents the minimum turning width of the tractor, m; *ψ* Tractor direction maximum angle, °.

The calculation formula of emergency turning acceleration is as follows:

3 $$\alpha_{\max } = \frac{{v_{{{\text{max}}}}^{{2}} R}}{1000} = {\text{g}}\varphi$$

where *α*_max_ represents the emergency turning acceleration, m/s^2^; *v*_max_ represents the maximum steering speed, m/s; *φ* ground adhesion coefficient.

2. Working condition analysis of tractor rear suspension farm tools

The tractor on the ordinary bumpy road normal at uniform speed, does not make the farm tool lift and farm tool lift, these two cases have different effects on the gearbox. When the rear suspension farm tool is lifted, as a part of the frame, the box is subjected to a remote vertical downward force. At this time, the box may produce bending deformation in the vertical direction, and even cause the box to break. The vector force can be defined as a part of the vehicle load in the static analysis. At this time, the lateral force is 0, and the box produces a downward bending deformation without torsional deformation.

3. Working condition analysis of a tractor when a tire is suspended

**Figure 5:**
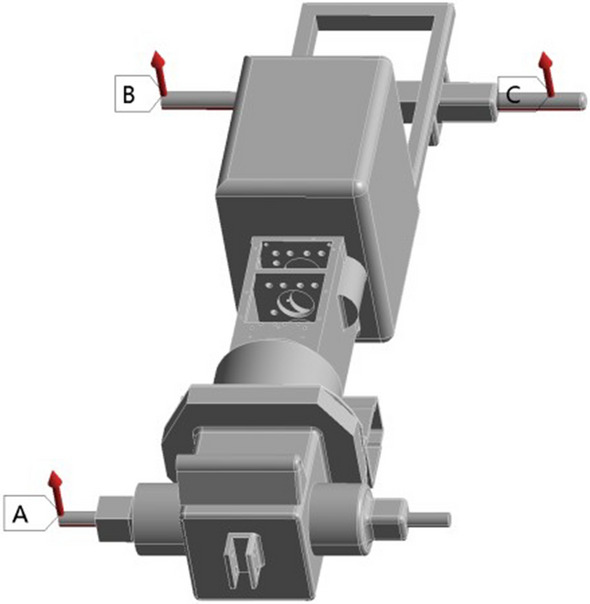
Simplification of box stress.

When working under harsh conditions, the tractor may have a tire suspension working condition. The box is squeezed by the engine and the rear axle connection bolt, resulting in bending deformation and lateral torsional deformation. The simplified model of the box and part of the frame is shown in Fig. 5.

When a tire encounters obstacles or pits, only three tires bear the weight of the vehicle, the maximum torque is determined according to the maximum carrying capacity of a single front wheel, and the lateral force is ignored.

4 $$Mn \, = \frac{{B_{1\max } Q}}{2}$$

Among them,

5 $$Q = \frac{{mg(1 - \lambda_{0} )}}{2}$$

Therefore,

6 $$M_{{\text{n}}} = \frac{{B_{1\max } mg(1 - \lambda_{0} )}}{4}$$

where *M*_*n*_ Torsion torque of gearbox housing, N·mm; *B*_*1max*_ Maximum wheelbase of the front wheel, mm; *Q* Maximum carrying capacity of the single front wheel, N; λ_0_ Vehicle quality distribution coefficient.

7 $$\tau = \frac{{K_{D} M_{n} }}{{W_{{\text{n}}} }} \le \left[ \tau \right]$$

where τ represents the torsional stress of the section, MPa; *K*_D3_ represents the load coefficient under torsion condition, taking 1.2; *W*_*n*_ represents the torsional section coefficient of the box section, mm^3^; [τ] Maximum allowable torsional stress, MPa.

ANSYS statics analysis of a wheel when applied to the torsional load should be under the requirements, the box generated torsional stress should meet the above formula calculation results^1^.

### Analysis of retaining condition

#### Gear force analysis

For plowing, subsoiling and other heavy load conditions, the gear load is high^17^. Using the rated torque 365 N·m of the engine, the torque transmission route of one gear and the basic parameters of each gear, the static load gear meshing force of the meshing gear at each axle bearing of the gearbox during one gear working is calculated.

Calculation formula of gear indexing circle diameter:

8 $$d = \frac{{{\text{z}} \times {{m_{n}}}}}{{{{\cos}}\beta }}$$

where* d* represents the diameter of the gear indexing circle, mm; *z* represents the number of teeth; *m*_*n*_ represents the normal modulus, *β* represents the spiral angle of the indexing circle^18^.

Calculation formula of gear meshing force:

9 $${\text{Tangential}}\;{\text{force:}}\,F_{\tau } = \frac{{{2}T{\text{d}}}}{{\text{d}}}$$

10 $${\text{Radial}}\;{\text{force:}}\;F_{\gamma } = \frac{{F\tau \times {\text{tan}}\alpha }}{{{\text{cos}}\beta }}$$

11 $${\text{Axial}}\;{\text{force:}}\;F_{\alpha } = F\tau \times \tan \beta$$

where *T*_*d*_ denotes the torque of the shaft acting on the gear, N·m.

#### Stress analysis of each fulcrum of box body

**Figure 6:**
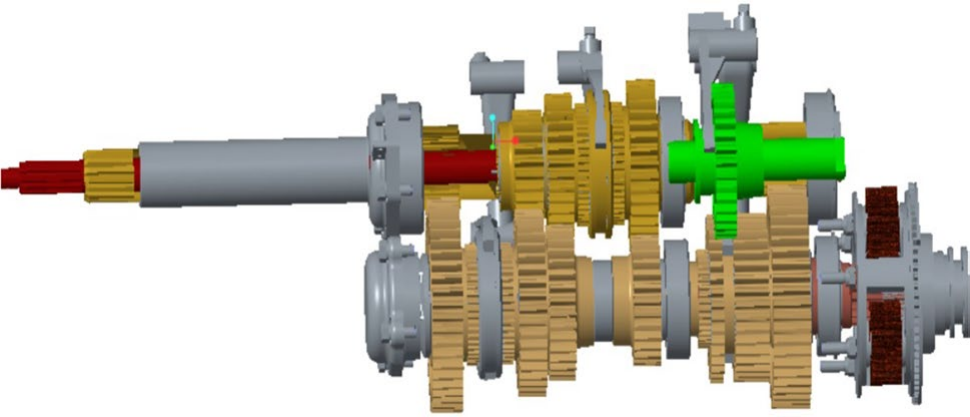
Three-dimensional diagram of transmission gear shaft.

The meshing force between gears is transmitted to the supporting bearing through the transmission shaft. The stress position of the gearbox box is the fulcrum at the contact between the box and the bearing, so the static load on the box is transmitted to the box through the bearing^19^. The gearbox contains input shaft, intermediate shaft, auxiliary transmission intermediate shaft, output shaft and inverted shaft, which are named S_1_, S_2_, S_3_, S_4_ and S_5_ respectively, as shown in Fig. 6.

**Figure 7:**
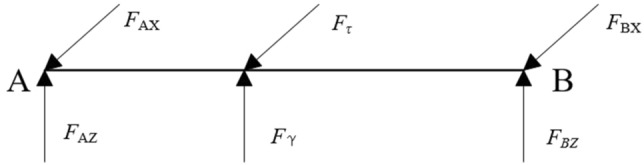
S_1_ axial force diagram.

The force calculation of each bearing is shown as Fig. 7.

According to Fig. 7, the force of S_1_ axis is balanced, so the balance equations are listed, and the unknown force at the bearing, namely, points A and C, is obtained.

12 $$\left\{ \begin{gathered} \sum {F_{X} } = F_{AX} + F_{{\tau_{1} }} + F_{CX} = 0 \hfill \\ \sum {F_{Z} = F_{AZ} + F_{{\gamma_{1} }} + F_{CZ} = 0} \hfill \\ \sum {F_{y} = 0} \hfill \\ M_{Azoy} = F_{{\gamma_{1} }} \times L_{AB} + F_{CZ} \times L_{AC} = 0 \hfill \\ M_{Axoy} = F_{{\tau_{1} }} \times L_{AB} + F_{CX} \times L_{AC} = 0 \hfill \\ \end{gathered} \right.$$

Similarly, the stress of S_2_, S_3_, S_4_ four shafts is calculated.

The components on each shaft are imported into ANSYS, and the corresponding material density is set. The mass of gears and transmission shafts is viewed in the Mass menu bar, and the total mass of each shaft is obtained. According to Formula (13), the gravity of each shaft is obtained.

13 $$G = mg$$

where *G* denotes gravity; m Represents quality; g Represents gravity acceleration, *g* = 9.8 m/s^2^.

By using the superposition principle, the self-gravity is superposed with the pressure in each direction of the bearing, then the force situation at each fulcrum is obtained.

After calculation, the force of the first support is shown in Table 1.

**Table 1:** Statement of support force of box.

Base	*F*_*x*_ (N)	*F*_*y*_ (N)	*F*_*z*_ (N)
1	10,208.00	0	3715.710
2	37,539.92	0	13,496.62
3	11,175.70	0	31,126.34
4	− 4736.72	0	− 12,447.59
5	− 18,794.36	0	− 12,636.80
6	− 32,616.89	0	− 12,018.81

Similarly, the stress of each base of the box under reverse working conditions is calculated, and the results are shown in Table 2.

**Table 2:** The force table of each fulcrum of the box body in reverse gear.

Base	*F*_*x*_ (N)	*F*_*y*_ (N)	*F*_*z*_ (N)
1	10,208.00	0	3715.71
2	37,539.92	0	13,496.62
3	11,175.70	0	31,126.34
4	− 4736.72	0	− 12,447.59
5	12,941.10	0	24,452.47
6	16,308.45	0	5851.38
7 (8)	− 11,959.53	0	− 4414.82

According to the above analysis, the vehicle condition and the gear condition are arranged and combined, and the following six conditions are divided as shown in Table 3.

**Table 3:** Simulation conditions.

Working condition division	Gear condition	Vehicle conditions
C 1	First gear condition	Lifting condition of rear suspension farm tools
C 2	First gear condition	There is a wheel suspension condition
C 3	Reverse gear condition	Lifting condition of rear suspension farm tools
C 4	Reverse gear condition	There is a wheel suspension condition
C 5	First gear condition	Emergency turning condition
C 6	Reverse gear condition	Emergency turning condition

## Static analysis and modal analysis

### Static analysis

#### Preprocessing

1. Import of box
  After Creo builds the model, save it as X_T format and open it in ANSYS Workbench.
2. Material setting
  Our research does not study the front axle, engine and rear axle housing. The material properties of the front axle, engine and rear axle housing are set to be rigid. The material settings of the box are shown in Table 4.
3. Grid division
  To have a good meshing quality, ANSYS Workbench automatic meshing method is used to divide tetrahedral meshes for this design, to avoid the inability to calculate due to excessive calculation. To ensure high calculation accuracy and moderate calculation amount, the grid size is defined as 10 mm, with slow and smooth transitions. The number of nodes is 334,884, the number of elements is 196,920, the average grid quality is 0.69562, the aspect ratio is 2.0493, the deflection is 0.29861, and the Jacobian is 1.0551. The grid division quality is good.

**Table 4:** Material data.

Box material	Poisson’s ratio	Elastic modulus/GPa	Density/kg/m^3^	Tensile strength/MPa	Yield strength/MPa
HT200	0.25	128	7000	510	350

#### Constraint conditions and load conditions

The setting of boundary conditions directly affects the accuracy of static analysis results. The six working conditions mentioned above correspond to six different boundary conditions, but the tractor as a whole system has many commonalities. When setting boundary conditions, these commonalities we need to reflect in each condition, after the actual survey, we set the following load conditions for the six conditions:

1. According to the equivalent mechanical model of the tractor, it can be seen that the front counterweight frame, cab and body structure, engine, gearbox, rear axle, auxiliary structure (fuel tank, etc.) are distributed on the transmission system and part of the frame. These structures are converted into remote uniform load in the form of equivalent weight and applied to the front axle, engine and rear axle connected to the box body, because these structures act directly or indirectly on the front axle, engine and rear axle, and then act on the gearbox box body, resulting in natural vertical bending deformation of the box body.
2. In the normal operation of the tractor, the counterweight is often set in front of the vehicle. Through the actual research and survey, we apply some body structure and the front counterweight block to the corresponding position of the front axle in the form of remote concentrated force, because the specific structure of the tractor is different, and the position of setting the remote force is also different according to the different models.

The six combined conditions have both commonalities and characteristics. It is found that when the tractor makes an emergency turn, if the ground friction coefficient can avoid the sliding friction of the tractor tire, the box will bear a large force to bend it. In the finite element statics analysis, we naturally constrain the rotational freedom around its central axis ' of the box, and apply an emergency acceleration to simulate the large centripetal force of the box when the tractor makes an emergency turn. When the tractor is lifting the farm tool, the box as a bearing member connecting the front and rear frames will be subjected to a remote force from the hanging farm tool, which we set in the position of the rear suspension center in the form of a remote concentrated force. When a tractor encounters a large pit or hard rock, there is often a dangerous situation of front wheel suspension, that is, a wheel suspension condition. At this time, the tractor's three tires support the frame structure, and the tractor frame and transmission structure bear a large torsional load. The such load can be achieved by setting constraints and load conditions.

The specific box constraints and load conditions are shown in Table 5.

**Table 5:** Boundary conditions of each working condition.

Conditions	Load conditions	Constraint conditions
C 1	The load is applied according to the force of each fulcrum in the first gear of Table 1 above; the gravity of the front counterweight, the gravity of the cab assembly and the maximum lifting force of the rear suspension farm tools are applied to their respective installation positions in the form of remote concentrated force^1^	The translational degrees of freedom in the three axial directions of X, Y and Z before and after the gearbox, as well as the rotational degrees of freedom around Y axis and X axis, are constrained, and then the rotational degrees of freedom around Z axis are constrained
C 2	The load is applied according to the force of each fulcrum in the first gear of Table 1 above; a 600 N · m torque is applied at the front axle engine, and the other loads are the same as the first condition	The translational degrees of freedom in the three axial directions of X, Y and Z before and after the gearbox, and the rotational degrees of freedom around Y axis and X axis are constrained to release the rotational degrees of freedom around X axis
C 3	The load is applied according to the force situation of each fulcrum under the inverted gear condition in Table 2 above; the vehicle load is the same as condition one	The constraint condition is the same as condition one
C 4	The load is applied according to the force of each fulcrum under the last working condition in Table 2 above; the vehicle load is the same as condition two	The constraint condition is the same as condition two
C 5	The load is applied according to the force of each fulcrum in the first gear of Table 1 above; here, a rotating acceleration of 0.8 is given to the gearbox housing, and other load conditions are the same as the working conditions	The translational degrees of freedom in the three axial directions of X, Y and Z before and after the gearbox, and the rotational degrees of freedom around Y axis and X axis are constrained to release the rotational degrees of freedom around X axis
C 6	The load is applied according to the force of each fulcrum under the last working condition in Table 2 above; other load conditions are the same as condition 5	The constraint condition is the same as condition five

### Modal analysis

A tractor gearbox can be regarded as a vibration system, and the reason for the vibration and strong noise of the gearbox comes from the sum of each excitation force applied to this system. The dynamic characteristics of mechanical structure mainly depend on its natural frequency, vibration mode and other modal parameters^20^. The natural frequency of the gearbox should stagger the inherent rotation frequency of the engine and the meshing frequency between the internal gear and the gear to improve the dynamic characteristics of the gearbox^21^. Main excitation sources of box vibration:

1. Engine rotational frequency
  Since the engine speed is variable, its vibration frequency is also variable, and the vibration frequency is in a region^22–24^. The rated speed of the tractor engine used in this design is 2300 r/min, calculated by the rated speed of the engine, the engine size, rotation frequency *f* = 39 Hz.
2. Meshing frequency between transmission gears
  The gear rotation and meshing process inside the gearbox is periodic, and this periodic motion leads to periodic vibration^22^. According to the continuous changes in the number of teeth, transmission routes and transmission speeds of different gears, the low-order frequencies generated by gears and their transmission mechanisms are mostly concentrated in 279.8–549.9 Hz^25^.

Modal analysis of tractor gearbox housing was carried out to test its vibration characteristics. Compared with unconstrained modal analysis, constrained modal analysis is more suitable for the analysis of the structure of the gearbox. According to the actual constraints, to simplify the constraints, and to constrain the degrees of freedom in six directions of the front axle, engine housing and rear axle connected to the box. Namely the translational degree of freedom in X axis direction, translational degree of freedom in Y axis direction, translational degree of freedom in Z axis direction, rotational degree of freedom around X axis, rotational degree of freedom around Z axis and rotational degree of freedom around Y axis.

## Results and optimization

### Test results of mechanical properties of box

#### The stiffness verification results

**Figure 8:**
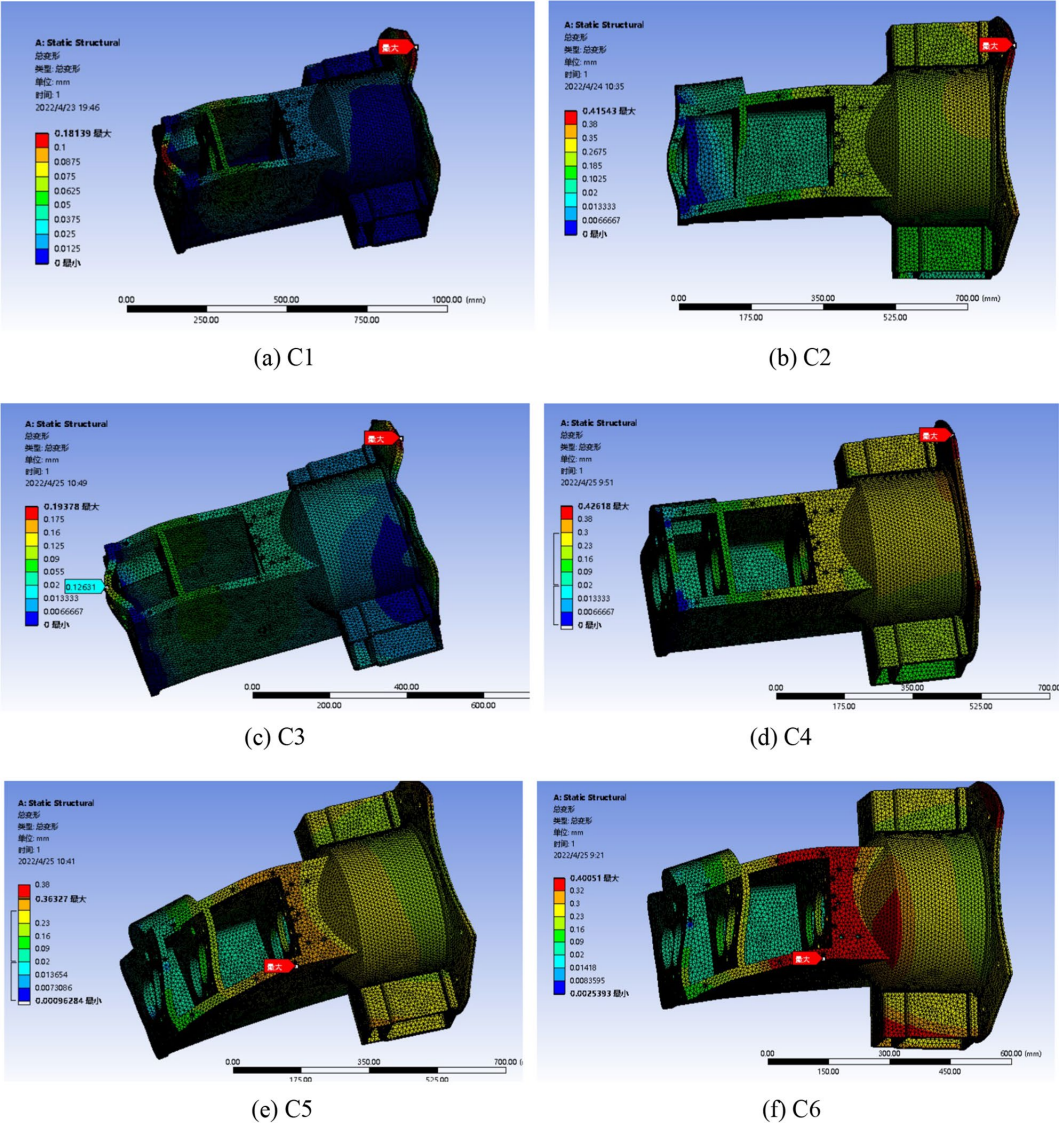
Deformation nephogram of the box under different working conditions.

The stiffness inspection of the gearbox housing structure is shown in Fig. 8(a–f).

By comparing the six working conditions of the above analysis, it can be seen that the deformation of the gearbox box is the largest when a wheel is suspended, and the deformation distribution is mostly concentrated at both ends of the box, mainly caused by the bending load exerted by the farm implements on the frame when the lifting farm implements. When a wheel is suspended and the gearbox works in reverse gear, the maximum deformation is 0.43 mm, which is less than the allowable deformation of 0.5 mm. The box meets the design requirements, but the maximum deformation is close to the allowable deformation. In the normal work of the tractor, there are bad conditions, which may lead to cracking or fracture of the gearbox box. By optimizing the structure and quality of the box, the deformation is reduced.

#### Strength check results

**Figure 9:**
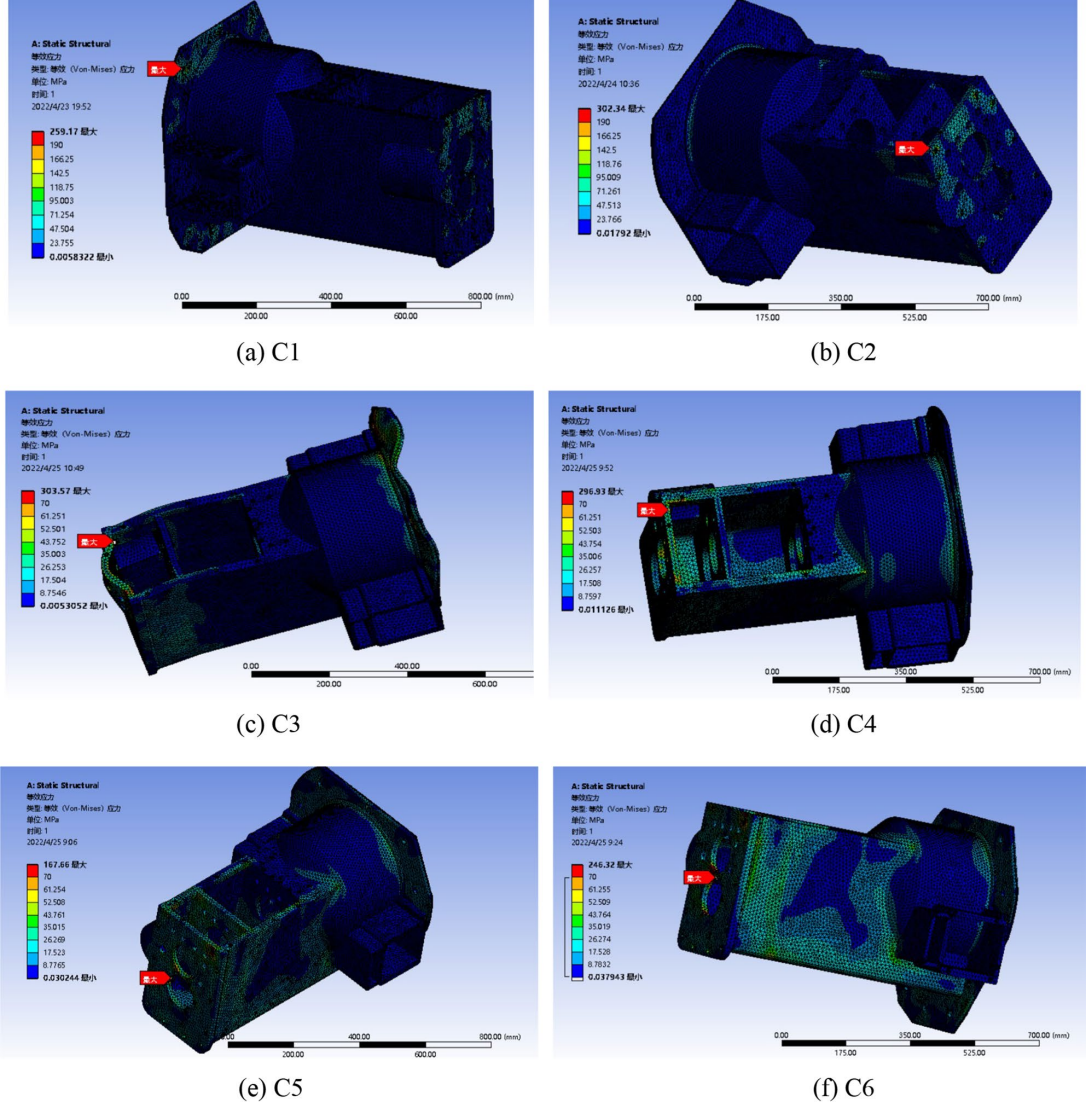
Stress nephogram of the box under different working conditions.

The structural strength check of gearbox housing is shown in Fig. 9(a–f).

By comparing the equivalent stress distribution of gearbox housing under the above six conditions, it can be seen that the vehicle condition has little effect on the stress distribution of gearbox housing. The maximum equivalent stress is on the wall of the box connected to the rear axle in the third case. Due to the existence of stress concentration, the equivalent stress value of the rectangular wall of the box is the largest, the maximum value is 303.57 MPa, the safety factor is 1.90, and the safety factor should be controlled between 1.2 and 2.5. The box meets the structural strength requirements by discarding the unremovable stress singularity, but there are still some stress concentration areas that may break. The stress concentration is reduced by optimizing the wall thickness and structure of the box.

#### Modal verification results

In ANSYS Workbench, the modal analysis module is used to carry out the constrained modal analysis of the box, and the sixth-order modal of the box is taken. The natural frequencies of the first six-order modal are shown in Table 6.

**Table 6:** Natural frequency table of sixth-order modes.

Modal number	Natural frequency (Hz)
1	651.4
2	726.72
3	821.21
4	895.63
5	994.13
6	1023.1

**Figure 10:**
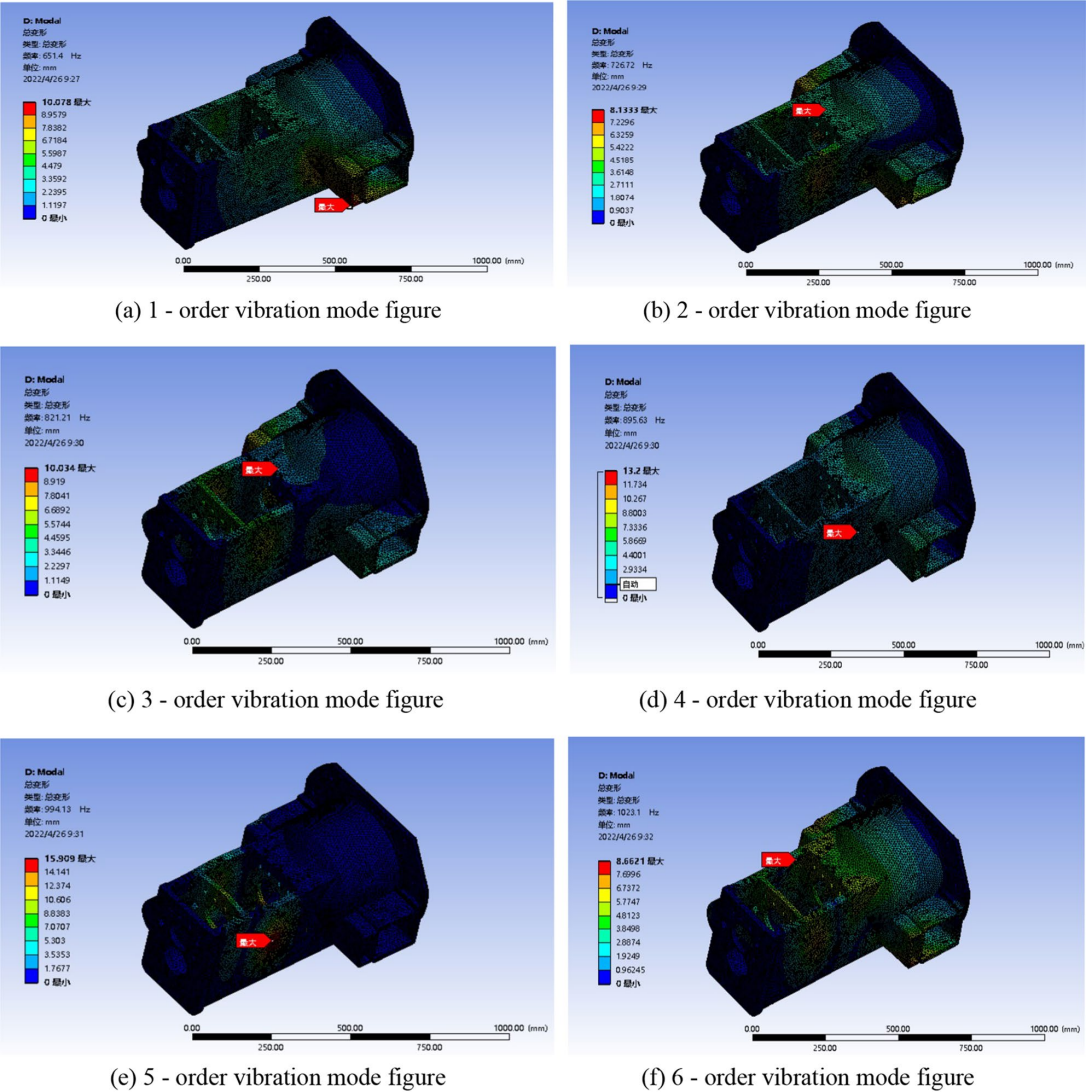
6-order modal analysis of gearbox housing.

The vibration mode diagram of the tractor gearbox box is shown in Fig. 10(a–f).

According to the results of modal analysis, the first six natural modal frequencies of the gearbox housing are a minimum of 651.40 Hz and a maximum of 1023.10 Hz, the frequency range is between 651.4 and 1023.1 Hz, and the periodic frequency of the gear meshing is between 284.4 and 550.5 Hz. At the same time, compared with the engine frequency of 2300 Hz, there is no resonance phenomenon. It is preliminarily concluded that the gearbox structure is reasonable, but the minimum frequency is close to the gear meshing frequency. The structure of the gearbox is optimized to maximize the natural frequency of the gear meshing frequency and the engine rotation frequency, and increase the structural safety of the gearbox.

### Box body optimizing

#### Optimization objectives and methods

1. Optimization objectives
  According to the structural strength test results of the box body, the deformation area of the box body which is more than the other parts is considered to increase the wall thickness, strengthen the reinforcement and the bearing beam to reduce the deformation; according to the stiffness test results of the box structure, the existence of stress singular points is identified and discarded, and the stress concentration area in the box structure is found. According to the results of modal analysis and actual use, the box model is improved, the box quality is reduced, the deformation is reduced, and the safety performance of the box is enhanced to achieve the purpose of optimization.
2. Optimization process
  The topology optimization module in ANSYS software can be used for topology optimization, automatic identification of box structure, quality and other aspects of defects or multi-system identification optimization process. It makes the structural material distribution of the gearbox more reasonable by reducing the design time^26^. It can make the box meet the strength, stiffness and vibration characteristics, reduce the box mass and optimize the material distribution of the box structure^16^. According to the results of topology optimization and static analysis, the structure and quality of the box are optimized and the stiffness and strength are checked to meet the requirements.

#### Topology optimization results

**Figure 11:**
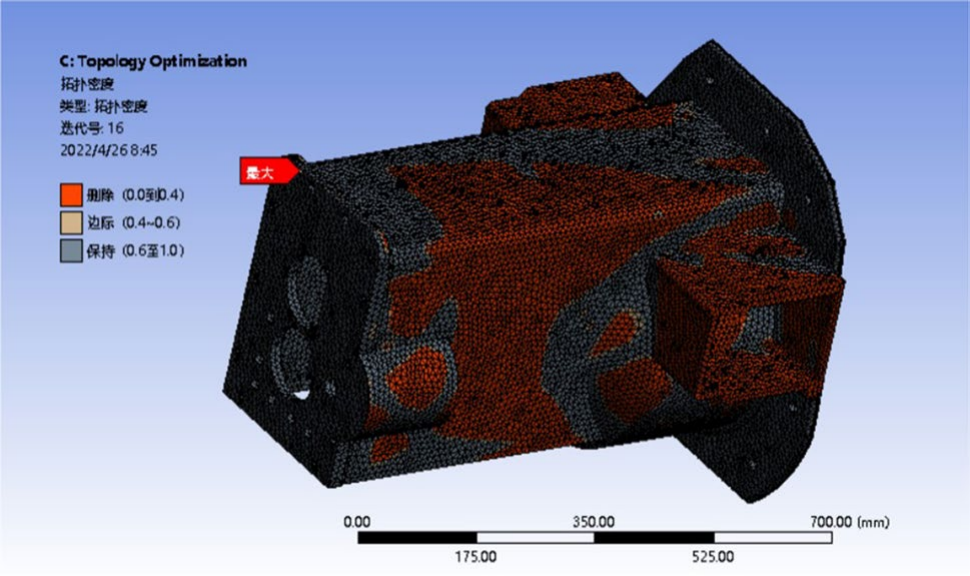
Optimization results with 50% mass reduction.

**Figure 12:**
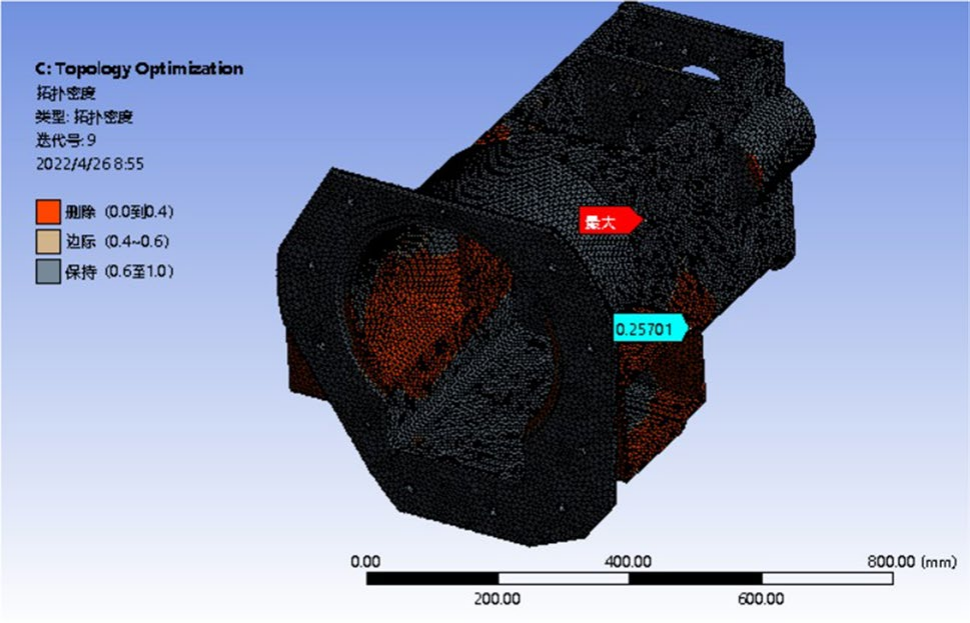
Optimization results with 20% mass reduction.

**Figure 13:**
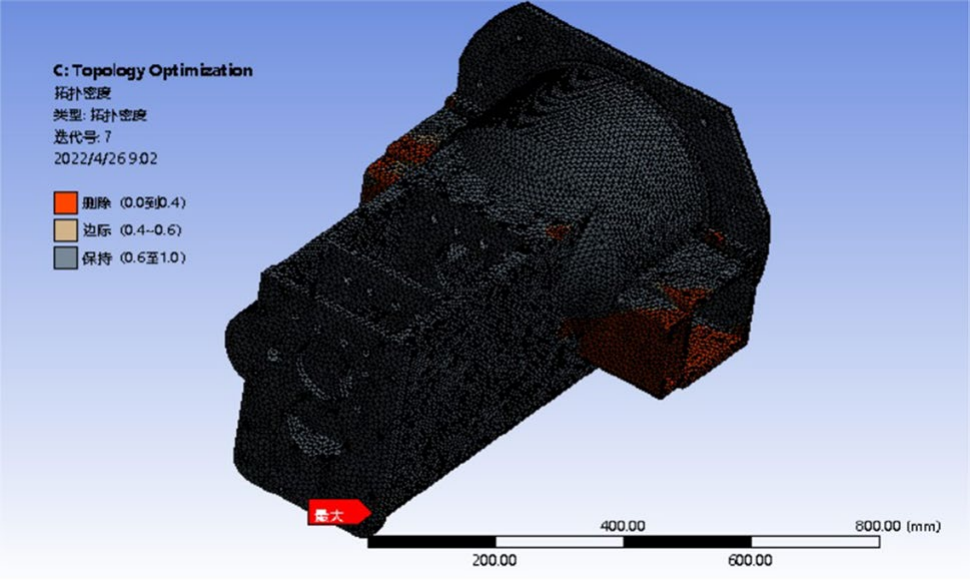
Optimization results with 10% mass reduction.

The third condition has a wide deformation distribution area and the most obvious stress concentration phenomenon, which can be used as a representative condition. So based on raw material setting and meshing, the load and constraint are simplified and applied under the three conditions. The iterative analysis is performed on 50%, 80% and 90% of the reserved parts, as shown in Figs. 11, 12 and 13. The analysis results are observed and recorded.

The red part in the diagram represents the part that the system defaults to remove, and the black part is the reserved part. The margin of the left yellow block in the figure represents the transition region of the box, which can be retained or removed^27^.

**Figure 14:**
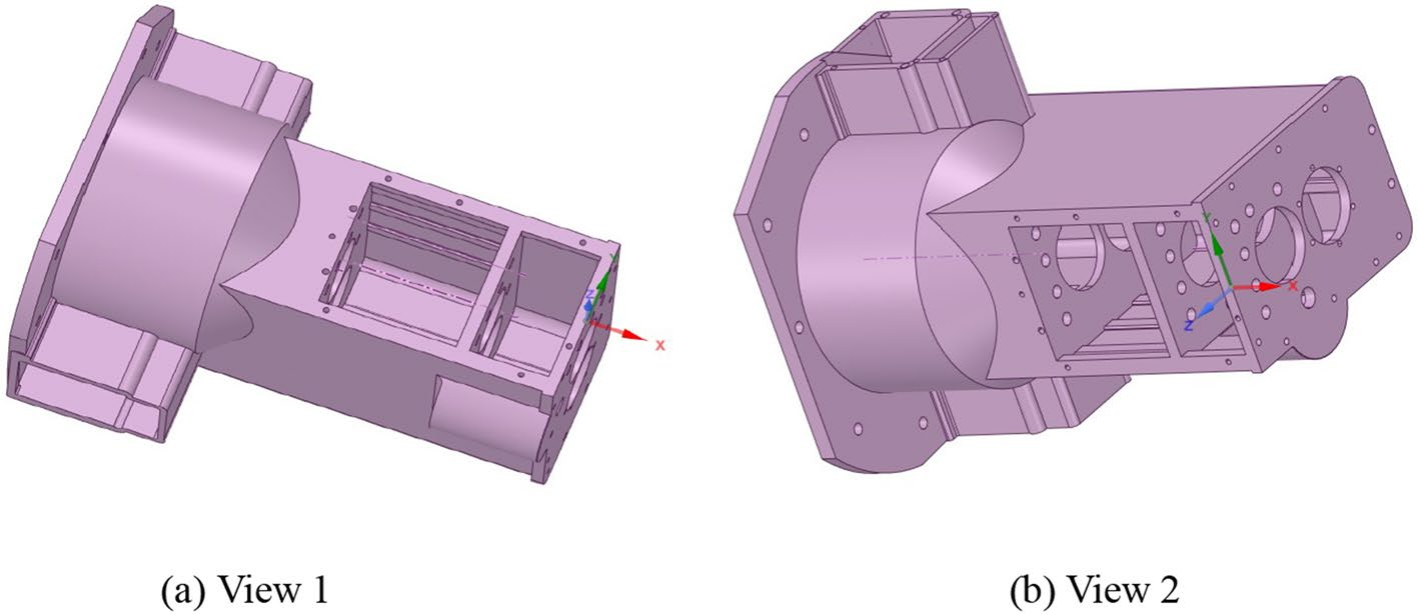
The optimized model of the gearbox.

It can be seen from the optimization results that the red area is mostly distributed in the box wall and the front surface of the front box, that is, the box wall thickness where the transmission shaft is located meets the requirements. Therefore, while ensuring the stiffness and strength of the box, the wall thickness here can be reduced to reduce the quality of the box. Both sides of the box are connected with the appendages, which can be removed under the premise of only reducing the weight of the box. The red area at the bottom of the box is less distributed, and the reinforcement or wall thickness can be increased. The box is divided into the front and rear boxes, the wall thickness of the front and rear boxes increases step by step, the wall thickness increases in the larger part of the deformation, and the wall thickness decreases in the smaller part of the deformation. The topology optimization under other working conditions is analyzed, and the original box is improved according to the analysis of the optimization results. The strength and stiffness of the optimized box are checked. The three-dimensional model of the optimized gearbox box is shown in Fig. 14.

### Analysis after optimization

**Figure 15:**
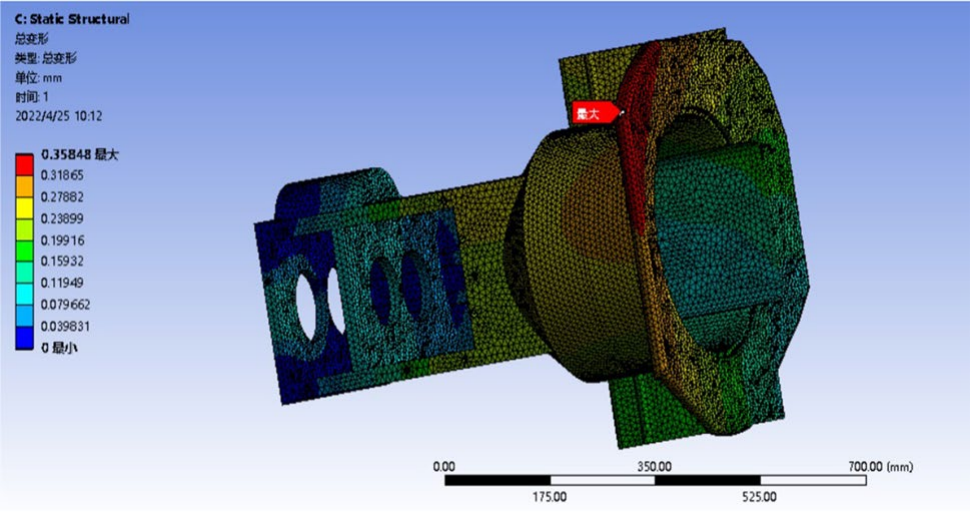
Four deformation nephogram of working condition.

**Figure 16:**
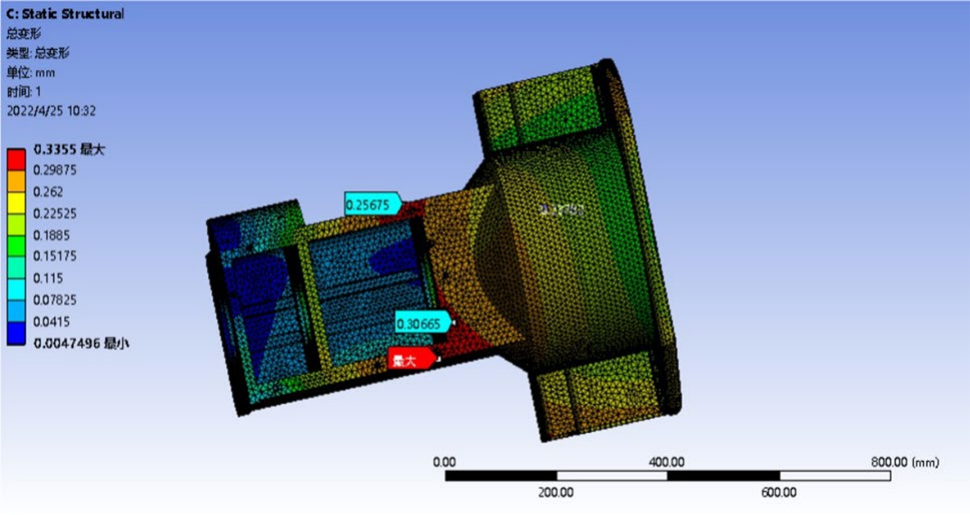
Six deformation nephogram under working condition.

**Figure 17:**
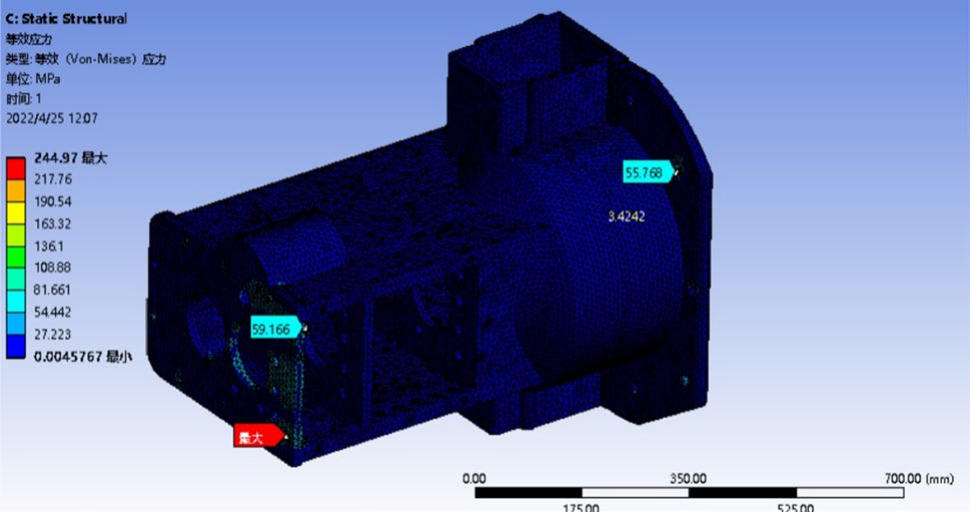
Three stress nephogram.

**Figure 18:**
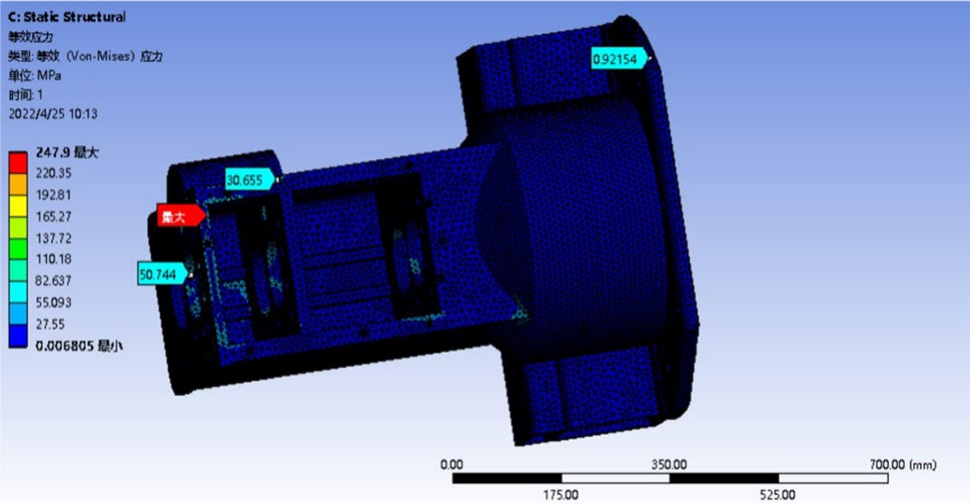
Four stress nephogram.

The optimized model only needs to analyze the maximum deformation or the most obvious stress concentration conditions, that is, the deformation analysis of condition 4 and 6, and the stress analysis of condition 3 and 4, and analyze the worst two conditions. The results of the strength analysis are shown in Figs. 15 and 16. The stiffness analysis results are shown in Figs. 17 and 18.

The comparison of parameters before and after box optimization is shown in Table 7.

**Table 7:** Comparison table of box optimization data.

Model type	Maximum equivalent stress	Maximum total deformation	Quality
Original model	303.57 MPa	0.42618 mm	139.79 kg
Optimization model	247.90 MPa	0.35848 mm	127.99 kg
Rate of decline	18.34%	15.89%	8.44%

According to the static analysis results of the optimized box and the data comparison results before and after optimization, the overall strength and stiffness of the box are significantly improved, and the deformation of the larger deformation is 15.89% lower than that of the original model, and the allowable maximum deformation of the box is less than 0.500 mm. The wall thickness on both sides of the box increases, and the stiffeners are set to reduce the torsional deformation of the box and improve the bending resistance of the box. After optimization, the stress concentration of the box is significantly reduced, and the inverted fillet is added in the stress concentration area, and the equivalent stress is significantly decreased. The maximum stress of 247.90 MPa is 18.34% lower than that of the original model. The rationality of the box structure is significantly improved to ensure the normal operation of the tractor gearbox under various harsh conditions. The box quality is reduced by 8.44% compared with the original model, and the strength and stiffness of the box are improved at the same time. These results show that the optimized box achieves the expected effect, and the improved box is more suitable for practical use.

## Conclusion

At present, there are some problems that the strength and stiffness of the tractor gearbox body cannot meet the allowable requirements. In extreme conditions, it may lead to box fracture or resonance phenomenon. It may lead to tractor rollover in severe cases. Based on the static analysis and modal analysis of the box, the topology optimization of the box is carried out to effectively improve the strength and stiffness characteristics of the box, to reduce the quality of the box, to reduce the waste of resources, to improve the safety coefficient of the gearbox, and to ensure the normal operation of the tractor under extreme conditions.

1. Based on the observation of the dangerous conditions that may occur when the tractor is working, six combined working conditions of the gearbox are analyzed. According to the load form and force size of the box under different working conditions, the boundary conditions suitable for each working condition are set. Restore the true condition of the box to the greatest extent through the setting of boundary conditions. Using the method of finite element static analysis, the structural strength and stiffness of the box are evaluated. The test steps are simplified, and the accuracy of box simulation is improved theoretically. The feasibility of the theory is verified by experiments.
2. On the premise of ensuring that the gearbox does not resonate, the topology optimization method is used to reduce the wall thickness of the side wall of the box with small deformation and thickness meeting the requirements. The final mass is reduced by 11.80 kg, which is 8.44% lower than the original model. The lightweight of the box is effectively realized, the waste of resources is reduced, and the heat dissipation performance of the box is improved.
3. According to the static analysis results of the gearbox, the maximum deformation and the maximum equivalent stress of the gearbox model before and after optimization are compared. It is obvious that the strength and stiffness of the gearbox are effectively improved. Compared with the original gearbox, the deformation of the improved gearbox decreases by 15.89%, and the maximum equivalent stress decreases by 18.34%. The stress concentration phenomenon is reduced, and the anti-deformation ability of the gearbox is enhanced, to ensure the normal operation of the gearbox under extreme conditions and prevent the occurrence of fracture.
4. The designed gearbox body can adapt to a variety of extreme conditions and ensure the normal shift operation of the gearbox; structural strength and stiffness meet the requirements of the use, and can effectively play a bearing cab and part of the framework function. Since the actual forging should be divided into the front and rear boxes, bolt connection is needed, and the structure is relatively complex, the box material should be biased towards lightweight setting.

## Data Availability

All data included in this study are available upon request by contact with the corresponding author.
